# Educational technology on tuberculosis: construction shared with Primary Health Care nurses

**DOI:** 10.1590/0034-7167-2023-0025

**Published:** 2023-11-13

**Authors:** Aloma Sena Soares, Laura Maria Vidal Nogueira, Erlon Gabriel Rego de Andrade, Élida Fernanda Rêgo de Andrade, Ivaneide Leal Ataíde Rodrigues

**Affiliations:** I Universidade do Estado do Pará. Belém, Pará, Brazil

**Keywords:** Primary Health Care, Family Nurse Practitioners, Tuberculosis, Educational Technology, Health Education, Atención Primaria de Salud, Enfermeras de Familia, Tuberculosis, Tecnología Educacional, Educación en Salud, Atenção Primária à Saúde, Enfermeiras de Saúde da Família, Tuberculose, Tecnologia Educacional, Educação em Saúde

## Abstract

**Objective:**

to develop, in a participatory way, an educational technology to assist nurses in the management of tuberculosis cases in Primary Health Care.

**Methods:**

methodological research with a qualitative approach. Data were collected between June and October 2022, in 25 Basic Health Units, with 41 nurses interviewed individually. Thematic content analysis was carried out to guide technology elaboration.

**Results:**

three empirical categories were organized, demonstrating the facilities and difficulties in tuberculosis management, the conceptions about educational technology as a facilitator of the teaching-learning process in Primary Health Care and participatory development of technology. Nurses were in favor of constructing an instructional guide technology, and made suggestions to encourage its creation and use in daily service routine.

**Final considerations:**

the participatory process made it possible to create technology to assist nurses in the teaching-learning process in Primary Health Care about caring for people with tuberculosis.

## RESUMO

**Objetivo:**

elaborar, de forma participativa, uma tecnologia educacional para auxiliar enfermeiros no manejo dos casos de tuberculose na Atenção Primária à Saúde.

**Métodos:**

pesquisa metodológica com abordagem qualitativa. Os dados foram coletados entre junho e outubro de 2022, em 25 Unidades Básicas de Saúde, com 41 enfermeiros entrevistados individualmente. Realizou-se análise de conteúdo temática para guiar a elaboração da tecnologia.

**Resultados:**

organizaram-se três categorias empíricas, demonstrando as facilidades e dificuldades no manejo da tuberculose, as concepções sobre tecnologia educacional como facilitadora do processo ensino-aprendizagem na Atenção Primária à Saúde e o desenvolvimento participativo da tecnologia. Os enfermeiros foram favoráveis à construção de uma tecnologia na forma de guia instrutivo, e teceram sugestões para incentivar sua criação e utilização no cotidiano dos serviços.

**Considerações finais:**

o processo participativo possibilitou criar a tecnologia para auxiliar enfermeiros no processo ensino-aprendizagem na Atenção Primária à Saúde sobre os cuidados à pessoa com tuberculose.

## RESUMEN

**Objetivo:**

desarrollar, de forma participativa, una tecnología educativa para auxiliar a los enfermeros en el manejo de casos de tuberculosis en la Atención Primaria de Salud.

**Métodos:**

investigación metodológica con enfoque cualitativo. Los datos fueron recolectados entre junio y octubre de 2022, en 25 Unidades Básicas de Salud, con 41 enfermeros entrevistados individualmente. Se realizó un análisis de contenido temático para orientar la elaboración de la tecnología.

**Resultados:**

se organizaron tres categorías empíricas, demostrando las facilidades y dificultades en el manejo de la tuberculosis, las concepciones sobre la tecnología educativa como facilitadora del proceso de enseñanza-aprendizaje en la Atención Primaria de Salud y el desarrollo participativo de la tecnología. Los enfermeros se mostraron a favor de la construcción de una tecnología en forma de guía instructiva y realizaron sugerencias para incentivar su creación y uso en el cotidiano de los servicios.

**Consideraciones finales:**

el proceso participativo posibilitó la creación de tecnología para asistir a los enfermeros en el proceso de enseñanza-aprendizaje en la Atención Primaria de Salud sobre el cuidado de las personas con tuberculosis.

## INTRODUCTION

Tuberculosis (TB), infectious disease caused by *Mycobacterium tuberculosis*, bacterium also known as Koch’s bacillus, persists today as a public health problem worldwide, due to its high incidence and mortality^(1,2,3)^. It is considered a global emergency that requires strategies for its control, through articulated actions involving epidemiological surveillance and organization of health services to decentralize access to early diagnosis, effective treatment of new cases and detection of bacterial resistance^(3,4)^.

Worldwide, in 2021, TB affected about 10 million people, leading to 1.4 million deaths^(5)^. In Brazil, 68,271 new cases were reported, equivalent to an incidence rate of 32.0/100,000 inhabitants. That same year, in the state of Pará, 3,711 new cases were notified, with an incidence coefficient of 42.6/100,000 inhabitants, and in the capital, Belém, 1,012 new cases were reported, with an incidence coefficient of 67.5/100,000 inhabitants^(6)^.

In recent years, although TB incidence in Brazil and in the world has been decreasing, it is crucial to recognize and intensify the participation of Primary Health Care (PHC) in TB management and control, in order to reduce incidence and mortality. The decentralization of detection, diagnosis, treatment and follow-up of TB cases to PHC contributes to guaranteeing the principles of universality, comprehensiveness and longitudinality within the scope of the Brazilian Health System (SUS - *Sistema Único de Saúde*), due to the proximity of the Health Units to the community and their role in ordering the Health Care Network (RAS - *Rede de Atenção à Saúde*) to prioritize the complexity of health care^(7,8,9,10)^.

In this scenario, the Family Health Strategy (FHS) is considered a key strategy to expand and strengthen PHC in the country, considering that it carries out actions to prevent illness, promotion and recovery of health in a decentralized manner, with the participation of the community and the presence of community health workers (CHW) in Family Health teams (FHt)^(8,10,11,12)^.

Thus, as TB control actions in PHC, the active search for respiratory symptoms (RS), an essential tool for early diagnosis, and the performance of directly observed treatment (DOT) stand out, which contributes to expanding therapeutic compliance, curing the disease and reducing treatment abandonment, also culminating in reducing the possibility of bacterial resistance to drugs^(4,8,9)^.

In this context, nurses’ role in FHS teams is observed in implementing the Tuberculosis Control Program, through managerial activities in service planning, organization and assessment, and care activities, as identifying RS, carrying out nursing consultations, reporting confirmed cases and requesting routine tests, among other actions that contribute to PHC’s resolving role in caring for individuals and their community, reducing the weaknesses involved in caring for people with TB^(13)^.

However, the effective control of the disease is a challenge for health teams that work in PHC, due to factors such as disorganization of the RAS, lack of motivation and lack of professional qualification. The network breakdown is related to the lack of laboratories available to perform diagnostic tests and medication supply failure, which make it impossible to fully guarantee DOT, factors that demotivate professionals. The lack or lack of professional qualification is related to the absence of permanent education, PHC professional turnover and unpreparedness in guideline and protocol management^(8,14,15,16)^.

The limitations inherent to professional qualification compromise service effectiveness and TB case management, since, in order to be successful in their activities, nurses must be able to carry out their work with awareness and safety^(8,14,17)^. Thus, it is essential to encourage the adoption of health practices based on scientific knowledge, which require efforts by professionals and managers. Among the strategies that direct and facilitate the teaching-learning process in the professional context, educational technologies (ET) stand out, tools that make it possible to inform and empower through appreciation of daily experiences and contributions of professionals in the educational process^(18)^.

The literature shows that ET use in the context of TB favors the teaching-learning process and the dissemination of technically adequate knowledge, also reiterating that these technologies are instruments of communication and health education^(19,20,21)^. Therefore, it corroborates the fact that ET promote the dissemination of knowledge and can encourage changes in attitude and behavior at different levels, contributing to qualify health care and add new perspectives for controlling chronic and infectious diseases such as TB^(22)^.

The participation of the target public in ET construction is relevant, as it allows valuing elements such as practices, knowledge and needs of people and groups, such as professionals, associating such elements with scientific knowledge to problematize and build joint solutions. Moreover, it favors the inclusion of new teaching-learning strategies to produce individual and collective knowledge, contributing to professionals’ autonomy as a transforming agent^(19,20,21,22,23)^. Thus, ET construction on TB in PHC can facilitate professional empowerment regarding prevention, diagnosis and treatment, making them co-responsible for actions to control the disease^(19)^.

From this perspective, despite the importance and contribution of ET to the teaching-learning process, the relationship between TB and ET is still little discussed in the scientific literature, especially when it comes to the participatory construction of ET with health professionals^(19,20,21)^. Thus, it demonstrates the need to invest in research with this approach to fill knowledge gaps, aiming at safe and effective nursing care.

The international literature reinforces the importance of better training professionals to properly detect and treat TB, revealing that using technologies in training activities can facilitate professional learning^(24,25,26,27,28)^. This has a relevant impact on health care quality, optimizing costs and time investment, in addition to contributing to expanding case detection and achieving the global goals of the End TB Strategy^(29)^. Therefore, streamlining teaching-learning actions can encourage professionals to comply with the necessary changes in care practice, considering the operational difficulties to carry out training activities on topics of collective interest, considering health teams’ work routine, which are often excessive^(30)^.

Despite official documents guiding nursing practice to control TB in Brazil^(31,32)^, there are locoregional disparities that limit the generalization of certain guidelines in the care for populations in the national territory^(33)^. In this regard, there is a need for an ET adapted to the local context based on the Ministry of Health recommendations and on scientific evidence presented with language and illustrations that contribute to reducing the variation of information and conduct offered by professionals to users. An accessible and dynamic tool with such characteristics can provide the professionals with the necessary tools, qualifying their actions.

It is understood that an ET in the context of TB, elaborated from nurses’ knowledge and needs, can mediate TB management between nurses and affected people, according to each social reality, and facilitate the process of continuing education among peers, contributing to expanding disease control based on safer and more qualified assistance. Carrying out a participatory/shared approach is recommended in the ET construction process, as it allows for interaction and exchange of knowledge, considering people’s needs and ways of life, thus presenting greater potential in its use^(22)^.

## OBJECTIVE

To develop, in a participatory way, an educational technology to assist nurses in the management of tuberculosis cases in Primary Health Care.

## METHODS

### Ethical aspects

The research was developed in accordance with Resolution 466/2012 of the Brazilian National Health Council. It was approved by the Research Ethics Committee of the nursing course at the *Universidade do Estado do Pará* (UEPA), and institutional authorization was obtained from the Municipal Health Department of Belém (SESMA - *Secretaria Municipal de Saúde de Belém*). All participants signed the Informed Consent Form (ICF) and, to protect their identities, were identified by means of an alphanumeric code, using the letter N, for “nurse”, followed by the sequential numbering of the interviews.

### Study design

This is methodological research, with a qualitative approach, guided by the COnsolidated criteria for REporting Qualitative research (COREQ) recommendations^(34)^.

### Study setting and data source

The research was carried out at 25 Basic Health Units (BHU) that have FHt in their setting, in the city of Belém, Pará, Brazil, representing 43.10% of the total of 58 BHU with FHt that make up the PHC service network in this municipality. Currently, Belém has 124 FHt, distributed among the 58 BHU.

A total of 41 nurses participated, 36.94% (41/111) of the total number of nurses working at FHS in the city at the time of the survey. Nurses who provided health care to people with TB for at least six months were included. Those who were away from their professional activities, for any reason, during the data collection period, were excluded.

Sampling was for convenience and, as a criterion to end the collection, data saturation was used, achieved when important new elements were not found and the inclusion of other information was not necessary to compose the data set and achieve the objective of the study^(35)^.

### Data collection and organization

Conducted by the main researcher, data collection took place from June to October 2022, organized in three stages: individual interviews with nurses; ET construction, according to the type defined by them; and ET introduction to nurses to approve the final version or suggest changes to qualify it.

In the first stage, the project was presented by the main researcher to nurses in their respective Health Units. This approach took place individually and, with those who agreed to participate in the study, interviews were scheduled, according to their availability and without interfering with professional activities. The interviews were carried out at nurses’ workplace, in reserved rooms at each BHU, ensuring comfort and privacy for participants.

A semi-structured script previously prepared by the researchers was used, containing open-ended questions about participants’ sociodemographic and academic-professional profile, their role in TB management and ET construction, in order to understand the knowledge and practices on the subject and direct the participatory construction of an ET. The interviews lasted an average of 20 minutes and were recorded in MP3 format after formal consent. With those who did not consent, responses were recorded manually.

### Data analysis

The content of the interviews constituted the *corpus*, submitted to the thematic content analysis technique^(36)^, taking into account its three stages: 1) pre-analysis, with text skimming to understand frequent expressions and themes in the testimonies; 2) material exploration, in which data were codified to identify context units, from which recording units emerged, qualified by the researchers as being the most frequent themes by occurrence and co-occurrence, proceeding to the grouping and categorization of these units through thematic element classification to identify the type of ET that professionals suggested as more appropriate and to organize the most frequent contents/themes, in order to compose the ET; 3) treatment of results, inference and interpretation, stage from which efforts were made to interpret and discuss the results in the light of relevant scientific evidence and the guidelines and recommendations contained in official documents on the subject.

Thus, three empirical categories emerged: *Facilities and difficulties in tuberculosis management in Primary Health Care*; *Educational technology as a facilitator of the teaching-learning process in Primary Health Care*; and *Participatory development of educational technology on tuberculosis in Primary Health Care*.

It is noteworthy that the ET will be submitted to validity processes of content and appearance and legitimization with the target audience in another study, in order to improve it and reaffirm its reliability and suitability for professionals. After that, it is intended to be made available online for download on a public access platform.

## RESULTS

### Participant characterization

Among the nurses, 87.80% (36/41) were female, aged between 26 and 59 years, with a predominance of the 26-35 age group for 56.10% (23/41). The years since graduation ranged from one to 30 years, prevailing the interval from one to five years for 41.46% (17/41). As for academic degrees, the majority (73.17%, 30/41) declared themselves to be specialists, predominantly outside the PHC concentration area (63.41%, 26/41) so that only 9.76% (4/41) mentioned specialization in the fields of collective health, primary care and/or family health (Table 1).

**Table 1:** Sociodemographic and academic-professional profile of participants, Belém, Pará, Brazil, 2022 (N=41)

Variables	n	%
Sex		
Female	36	87.80
Male	5	12.20
Age		
26 to 35 years	23	56.10
36 to 45 years	10	24.39
46 to 55 years	6	14.63
≥ 56 years	2	4.88
Years since graduation		
1 to 5 years	17	41.46
6 to 10 years	15	36.59
11 to 15 years	5	12.20
16 to 20 years	2	4.88
≥ 21 years	2	4.88
Academic background		
Undergraduate degree	11	26.83
Specialization	30	73.17
Area of specialization		
Collective health, primary care and/or family health	4	9.76
Other	26	63.41
Tenure at the BHU		
< 1 year	6	14.63
1 to 2 years	10	24.39
3 to 4 years	16	39.02
5 to 6 years	4	9.76
7 years	5	12.20
Length working in care for people with TB		
< 1 year	2	4.88
1 to 5 years	22	53.66
6 to 10 years	13	31.71
11 to 15 years	1	2.44
16 to 20 years	3	7.32
Source of specific training to work with TB cases		
SESMA	35	85.37
Did not receive training	6	14.63
Last training		
1 year ago	25	60.98
2 years ago	3	7.32
3 years ago or more	7	17.07
Did not receive training	6	14.63

Tenure at the BHU ranged from one month to seven years, with an interval of three to four years prevailing for 39.02% (16/41), and the length working in caring for people with TB ranged from six months to 20 years, with 53.66% (22/41) working from one to five years. With regard to specific training to work with TB cases, the majority (85.37%, 35/41) reported that they were trained through SESMA. In this context, 68.29% (28/41) stated that they had participated in some improvement or update program on TB in the last two years (Table 1).

Below are the empirical categories generated by qualitative data analysis, from which the ET was elaborated.

### Facilities and difficulties in tuberculosis management in Primary Health Care

In this category, the factors that facilitate or hinder TB management in nurses’ daily activities stand out.

Regarding the factors that they consider facilitators of their care practice, the following were observed: the recognition of FHS and the presence of CHW as essential for disease control; the technical-scientific knowledge and FHS nurses’ autonomy in caring for people with TB as well as the close relationship between PHC professionals; the municipal coordination of TB control and referral services, facilitated by resources that enhance interpersonal communication, such as WhatsApp^®^:

*The issue of us working at the FHU* [Family Health Unit, referring to one of the facilitators] *and having* [community] *health workers to search for them* [patients with TB], *to follow up properly, that makes it much easier.* (N2)

*We end up having very good clinical knowledge, so we are able to identify when that patient has signs and symptoms of tuberculosis, even before he undergoes a specific test.* (N41)

*WhatsApp* ^®^ *contact makes it very easy. All the doubts that I have, regarding the patient, that I think I am not firm in what I am going to do, I just send* [messages through] *WhatsApp* ^®^ [to the professionals of the municipal coordination of TB control] *and in the same time they answer me.* (N6)

Regarding the difficulties that may influence their care practices for people with TB, operational issues predominated, such as limitations in medication supply in the basic therapeutic scheme for immediate initiation of treatment, which should always be available in services. Professionals associated these limitations with three main factors: the need to order medications from the municipality’s warehouse and an employee of the Health Unit to transport them to the service, making this supply logistics expensive and time-consuming; lack of adequate physical structure to care for people with TB, as some rooms are poorly ventilated, do not have windows and have indoor cooling devices; delay in delivery of bacterial culture results with sensitivity test.

An explicit difficulty in the testimonies concerns the centralization of TB control actions on nurses, mischaracterizing the multidisciplinary team work for people with TB, as recommended by the Ministry of Health. This reality of services in the municipality leads to overload of nurses and compromises action quality.

Another aspect is the lack of specific knowledge, norms and guidelines of the TB Control Program on case management, indications for laboratory tests and the need for assessment and care for patients in secondary or tertiary care, compromising action and patient care quality:

*We, who do all the treatment* [referring to nurses], *have to ask for medication, request sputum control tests. During the entire treatment, the patient spends only with us. If we see that we need to see the doctor, we already request an appointment with the doctor. But the treatment itself is under our responsibility, even if another colleague has detected it. And then, in this case, I think this responsibility could be divided, because, the rest of the things, everything is also under our responsibility.* (N3)

*Exams, when to order? For instance, how often to ask, is something I still have a little doubt about. I never needed, for instance, to request RMT* [rapid molecular test for TB], *but I would not know how to request it, I think I would have to open it there* [a Ministry of Health manual] *to read when the indication is.* (N41)

*I have doubts about the patient who abandons. There are patients who abandoned treatment three times, so, in relation to that, I have doubts whether* [the professional] *can refer the patient to the Referral Hospital or if he stays in PHC.* (N11)

### Educational technology as a facilitator of the teaching-learning process in Primary Health Care

In this category, nurses’ conception and practices regarding permanent education and their perception of ET use as a resource that enhances and facilitates the teaching-learning process in PHC are presented.

Unanimously, it was found that nurses understand the importance of continuing education to qualify PHC services. However, they reported difficulties in implementing educational activities due to three factors: lack of physical space and adequate resources to carry them out; limitations in planning and organizing training given the volume of activities under their responsibility; and operational barriers to bring together a specific or comprehensive group of professionals, in order to present and discuss certain topics of individual and collective interest.

Bearing that in mind, they reported that it is more frequent to carry out updates only when the municipal health management schedules them, prioritizing certain professional categories. In Health Units, it is more common to provide impersonal and individual guidance to a portion of team members, such as CHW and nursing technicians, on subjects they refer to and/or about which they observe that there is some lack of knowledge:

*It’s more about the issue of time* [explaining the difficulties], *because, with many things, nurses are responsible for practically everything at the* [Health] *Unit, and it is a little difficult for us to sit down to schedule a lecture, a training course, this is already more difficult.* (N3)

*We are not doing* [permanent education activities] *because of space. We only have one office* [in the Health Unit], *there is no space to do this education.* (N11)

*We don’t have time, we’re always busy with other* [health care] *programs. We meet when we see the need to talk* [about] *some subject or if there is any update. Generally, I guide them, individually, when I see that they are having some difficulty. They come to me to clarify doubts.* [About this]*, we deal more through WhatsApp* ^®^. (N33)

When asked if ET use could be useful for educating professionals, the perception was unanimously identified that this could be a mediating instrument of educational processes in nurses’ experience in PHC, referring that the ET facilitate the understanding of certain subjects in a clear and objective way because they are didactic resources, and allow their use for consultations with the material after professional training and/or updating:

*Yes, because it improves the explanation of a subject, improves understanding and, consequently, improves patient care.* (N1)

*For sure! I like to use the technologies they illustrate, because we manage to use two forms of visualization, of fixation, both my speech and, for instance, the analysis of the drawing or a material that I can give to them* [professionals target of educational activities] *read. So, this makes it easier for them* [professionals] *to absorb the information.* (N17)

*It helps, because we work with the visual, in a direct way, it is not difficult to understand. And it standardizes the information, everyone knowing the orientation and the correct flow, guides the professional and everyone helps each other.* (N29)

### Participatory development of educational technology on tuberculosis in Primary Health Care

In this category, professionals’ suggestions are presented regarding the ET form and content to be used in routine services.

Regarding the type of technology, *a priori*, it was observed that opinions were divided between the development of materials or tools such as a folder, booklet, guide, training program, electronic platform/interactive website or mobile application. The relevant aspect for choosing the type of ET was the difficulty in accessing the mobile network and the internet, which is not available in some units as well as the possibility of consulting the material at the time of nursing consultation, both in the form of digital media with files inserted in electronic devices for personal use, such as cell phones and tablets, and in printed form.

From this context, it was considered more appropriate to prepare an ET in printed form, also available in digital file in PDF format, prepared through Canva, a free online graphic design platform:

*A booklet by your side, a reference book, because we work with several* [health care] *programs, we do several things at the same time, so it would be good to have a kind of guide for us, a reference tool for the nurse, because sometimes I forget* [certain details]. (N8)

*An online document would be very interesting as well. So, I think the amount of paper should be reduced more, because, nowadays, everyone lives on their cell phones. You picked up your cell phone and you’re already reading, you’re already studying.* (N6)

*I think it would make it easier if we had access to the protocols, to these documents in hand, like, printed, because, here in our reality, we have a lot of difficulty with the internet issue.* (N3)

As for ET content, it was considered pertinent to produce synthetic, easy to understand and easy to visualize material, which addressed everything from the detection of suspected cases of TB in the community, going through aspects such as diagnosis, indication of each laboratory test, treatment, guidelines for people with TB, clinical management of cases of therapeutic abandonment and adverse drug reactions, assessment of contacts, flow of care in the city’s care network and nurses’ role in TB case management:

*How to detect patients in the community* [where] *you go, how to identify patients, how to guide the community, how to diagnose, how to care, treatment, adverse reactions.* (N8)

*When do I use the* [bacterial] *culture? What type of patient will I need to order a culture or RMT? Sometimes, we do not know when each type of exam is indicated.* (N40)

*It could be a document focused on treatment, on conducting the exams, how to assess the exams, that would make it easier. Not only the issue of TB in general, but also, for instance, LTBI* [latent tuberculosis infection], *differentiations, how to manage? I carried out the first analysis with the laboratory test, how to conduct these six months* [of treatment] *from now on? Even communication with the secondary area sector, for instance, “Do I need a secondary* [care] *assessment or not? Or right here* [at PHC], *can I do it?”. In the sense of from when do I forward? You need to have the introduction of these flows clear, because they are not clear,* [...] *where should I forward them? I’m feel lost.* (N4)

After elaboration, the ET was presented to 61.00% (25/41) of nurses, since it was not possible for everyone to participate in the period foreseen for ET introduction to participants. These were divided into two groups: seven participated in synchronous virtual meetings via Google Meet^®^, being developed with four professionals in the afternoon shift and three in the night shift, and 18 received the ET by email. Thus, they were able to appreciate, assess and indicate adaptations of content and design. They approved the material, and considered the general context of the information and illustrations to be clear and relevant. Some made small suggestions for adjustments in relation to the text, which were accepted by the researchers.

After the changes, the final version of the ET was elaborated, entitled “*Guia sobre tuberculose para enfermeiros da Atenção Primária à Saúde de Belém do Pará*”, in brochure format, with 18 color pages. Page layout and diagramming were carried out in horizontal orientation in standard formatting size, approximately 11 cm high by 20 cm wide, consisting of textual content and illustrations. The titles were formatted with font *Linus Biolinum*, varying in sizes between 20 and 36, and the texts, with font Assistant Regular, varying in sizes between 20 and 24.

The guide features a cover page, presentation page, 15 sections related to the most recurrent participants’ doubts on TB management and a list of references with official publications, especially from the Ministry of Health. These 15 sections address: RAS in the city of Belém on care for people with TB; identification of a suspected TB case; flowchart for welcoming a suspected TB case; indications for laboratory tests in the context of TB; flowchart for diagnosing and monitoring TB cases; TB treatment regimen for people aged over 10 years; guidelines for people undergoing TB treatment; special situations in the treatment of people with TB; follow-up of cases under treatment; adverse reactions to treatment; management of cases under treatment; flowchart for person who interrupted treatment; LTBI investigation; evaluation of contacts of people with TB aged 10 years or older; LTBI treatment regimens.

Thus, in order to develop an ET, we sought to know nurses’ reality and identify their knowledge and practices on TB management, in order to contribute to the care for people with this disease in PHC. It is noteworthy that participants were in favor of ET elaboration in the form of an instructional guide, and brought relevant suggestions to encourage its creation and use in the daily life of services.

## DISCUSSION

Shared ET construction was essential to elaborate a material adequate to professionals’ needs and reality, since it will be used by them. The recognition of PHC capillarity, through FHS, and the importance of CHW in the active search for RS as well as the respective control of the disease by the professionals who participated in this study are in line with the Brazilian National Program of Tuberculosis Control (PNCT - *Programa Nacional de Controle da Tuberculose*) guidelines and recommendations, advocated by the Ministry of Health to decentralize and horizontalize TB surveillance, prevention and control actions in PHC^(10)^.

Strengthening PHC to achieve universal health care is the best strategy to control the disease, given the proximity of the first level of care to the community. Therefore, it is essential to guarantee universal access to health services for the entire population, with a view to the needs and specificities of each population group as well as investing in public health with health professionals’ qualification to properly manage the disease and the structuring of PHC services, incorporating technologies necessary to establish diagnosis and treatment in a timely and early manner, in order to improve epidemiological and operational indicators and contribute to eliminating TB^(7,15,37)^.

It should be noted that the decentralization process does not occur homogeneously in the country, due to the lack of structural resources and obstacles in the articulation between services, reflecting on TB control^(38)^. In this scenario, major challenges are faced by PHC, many of which were reported in this study, such as the absence or lack of infrastructure and organization of services in RAS, maintenance of care activities with qualified professionals and work overload for nurses, situations that, similar to this result, are corroborated by different studies^(7,^8,39^,40)^.

Medication supply, carrying out laboratory tests, following specific therapeutic protocols for treatment and installation of adequate physical spaces for case care are essential factors to implement a set of actions that will provide the effective TB control, since, with the disposition of these factors, actions occur more effectively in PHC^(3,38)^. Still in this perspective, the installation of airy and adequate offices, where nursing consultations and certain reception activities can take place, should compose the structure of BHU and be inherent to the care processes that contribute to strengthen disease management^(38)^.

Other important data relate to the lack of shared care for people with TB and failures in communication between team professionals, also found in surveys carried out in the city of Belém, Pará^(39)^, and in the city of Meleiro, Santa Catarina^(40)^, which identified a limited partnership between nurses and other health professionals in caring for people with TB, a fact that can directly influence access and compliance with treatment. Communication weaknesses in FHS teams tend to cause work overload for professionals in certain categories, especially nurses, because the work processes in this health care model require, from those who work in it, skill and capacity to establish fruitful interpersonal relationships, aiming at a more resolute health care^(40)^.

Bearing this in mind, it is understood that most of the problems in TB control reside in the way in which health services are organized to detect and treat cases. Thus, to qualify care activities from detection, reporting and follow-up of cases, using therapeutic protocols and safe intervention strategies, it is fundamental that there are initiatives of permanent education about TB in PHC to strengthen multidisciplinary work in care networks. The implementation of these initiatives implies developing new care tools in health services, corroborating or redefining roles, responsibilities and action strategies for effective TB control^(38)^.

Despite being an old disease, knowledge gaps and inadequate attitudes and practices for TB control and prevention still permeate actions among health professionals around the world, contributing to increase the risk of infection, maintain the chain of transmission and generating negative impacts on individual and collective daily life^(41,42,43)^. Thus, training methods that are accessible and appropriate to the social reality, which optimize time and motivate professionals, can facilitate the understanding of the problem and bring about changes in health work, reflecting on higher quality care^(30)^.

From this perspective, recognizing nursing role in coping with TB and the importance of tools that support nurses in managing the disease in PHC services, the Ministry of Health published, in 2021, a guiding instrument that strengthens professionals’ autonomy in their work processes and favors the teaching-learning process, considering that it presents, with didactic and explanatory resources, different theoretical and operational aspects such as disease diagnosis and treatment, TB/HIV co-infection, LTBI, social protection mechanisms, care and counseling for people with TB^(31)^.

This instrument points to standardized and quality health care throughout the country, supporting decision-making and clinical nursing conduct^(31)^, similarly to the guide presented in this study, which, in turn, displays and values local particularities to assist nurses in TB case management in the RAS of the municipality of Belém, since it was developed in a shared way. In view of this, both tools are congruent with evidence that reaffirms the need for a dynamic, autonomous, safe and representative training process for nurses^(17,23,26)^.

In the scope of health work, permanent education corresponds to the educational processes that incorporate teaching and learning into the routine of services, enabling critical-reflective analysis and production of knowledge about social reality. However, it is necessary that the training process be planned according to the specific reality of each service, considering regionalization and the agents involved in this process, in order to favor the resolution of issues involving the health sector, contributing to expand and strengthen SUS’ resolving capacity and effectiveness^(44,45)^.

Among the various strategies to carry out educational processes with the professionals who work in the services, ET use is increasingly frequent, in order to contribute to their training and updating, considering that professional development is essential to maintain or improve service quality. Thus, it is understood that ET, especially those constructed through participatory research, have a potentially transforming nature of the reality experienced by nurses in PHC, because, based on the needs that arise from professional routine, they can mediate meaningful learning, enabling the construction of knowledge combined with knowledge and practices acquired through professional experience^(46,47)^.

In this context, the participatory elaboration of ET presented in this study aimed to promote professionals’ involvement to meet the demands of learning in the care for people with TB and, thus, collaborate with controlling the disease from the multilateral opportunities offered by PHC. Accordingly, the literature reveals that the most appropriate technologies to assist in the teaching-learning process in health are those printed with illustrations, which are easy to understand and present content according to the target audience’s educational and cultural level, favoring dissemination of knowledge, generating skill and autonomy^(22)^. Thus, the type of ET chosen by the participants of this study presents itself as a viable alternative to mediate professional training.

Carrying out health promotion actions in PHC and building an ET to assist in the work process of those who work in it are challenging efforts. However, despite the challenges, it is important to emphasize that health education in PHC is a tool that aims to qualify individuals’ and groups’ knowledge, attitudes and practices, such as professionals involved in provision of care, helping to promote the health of those they care for. The incorporation of educational materials in nursing professionals’ activities is already frequent and makes health work more productive and safer, facilitating the orientation processes directed to other professionals or to the population assisted by services, as they mediate professional practices^(48)^.

Thus, having educational and instructive material on TB facilitates and standardizes the essential guidelines for nurses, with a view to health care, providing new experiences within the processes of acquisition and production of knowledge, about oneself about one’s needs and/or the context in which one is inserted. When proposed in this perspective, the educational material can develop the necessary potential to help in professional routine, as it will serve as a source of information about health care^(22)^.

### Study limitations

As a limitation, it is considered the fact of not being able to count on all participants in the ET assessment stage, due to the incompatibility of some professionals’ schedule, considering that the participation of 100% of them could contribute to better qualify the product. Despite this, it is understood that the results were sufficient to support the process of shared construction and endorse the ET.

### Contributions to nursing and health

ET elaboration is an advance for health education activities, as it is an easy-to-apply tool, based on the learning demands of professionals who work in TB control, and can be used to enhance the teaching-learning process in daily health service routine, considering the participatory approach of ET.

Moreover, it can contribute to disseminating technically grounded, updated and easily assimilated information by professionals, resulting in the improvement of comprehensive care quality for people with TB and in disease control, with consequent repercussions on morbidity and mortality rates.

## FINAL CONSIDERATIONS

ET creation in the form of a guide demonstrates the potential use of this tool to qualify nursing actions in the care for people with TB, due to the joint participation of professionals during its construction. In this regard, it is proposed that ET assist in care activities and guidelines shared in professionals’ daily lives, given their recognition that ET is an important resource for the teaching-learning process in PHC and can strengthen work in health, especially in care and management activities, often undertaken by nurses.

It is emphasized that permanent education is a guiding tool for qualified assistance in coping with TB, necessary in the context of PHC and RAS, and can be operationalized through strategies such as ET. Therefore, there is a need to invest in more research like this one, aiming to strengthen and qualify PHC and TB control. Still in this perspective, it is relevant and opportune for the study to proceed to the validity stage of the technology developed, in order to reaffirm ET reliability, facilitate its wide insertion in health services and thus mediate PHC professionals’ training processes through its use.
